# Measuring the Activity of Mental Health Services in England: Variation in Categorising Activity for Payment Purposes

**DOI:** 10.1007/s10488-019-00958-7

**Published:** 2019-07-27

**Authors:** Rowena Jacobs, Martin Chalkley, Jan R. Böhnke, Michael Clark, Valerie Moran, M. J. Aragón

**Affiliations:** 1 Centre for Health Economics, University of York, Alcuin A Block, Heslington, York YO10 5DD UK; 2 School of Nursing and Health Sciences, University of Dundee, Dundee, UK; 3 Care Policy & Evaluation Centre, London School of Economics & Political Science, London, UK; 4 Luxembourg Institute of Health and Luxembourg Institute of Socio-Economic Research, Luxembourg City and Esch-zur-Alzette, Luxembourg

**Keywords:** Mental health funding, Provider payment, Episodic payment, Variation, Costs

## Abstract

In the context of international interest in reforming mental health payment systems, national policy in England has sought to move towards an episodic funding approach. Patients are categorised into care clusters, and providers will be paid for episodes of care for patients within each cluster. For the payment system to work, clusters need to be appropriately homogenous in terms of financial resource use. We examine variation in costs and activity within clusters and across health care providers. We find that the large variation between providers with respect to costs within clusters mean that a cluster-based episodic payment system would have substantially different financial impacts across providers.

## Introduction

There is significant international interest in developing payment approaches in mental health services that create the right incentives to use scarce resources more efficiently without compromising care quality (Moran and Jacobs 2017). Public providers of mental health services in England (National Health Service (NHS) Trusts) have historically been funded through block contracts agreed between commissioners (who purchase care services) and providers of care, and usually this has been on the basis of levels of existing ‘inputs’ such as the number of beds (Mason et al. 2011). This method of financing offers little financial incentive for providers to efficiently meet health care needs and can have perverse incentives for quality of care (Jacobs 2014).

For England, starting in 2012 the Department of Health mandated that mental health services move away from block contracts and use newly developed *care clusters* to classify patients according to their needs, with payment related to episodes of care within a cluster, an approach referred to as *episodic payment* (Department of Health 2010, 2013). Implementation of the use of the clusters and development of payment models has largely been devolved to local commissioners and providers of mental health care. Subsequently, there has been much debate about the use of the clusters with hope that the approach had merit, but also caution that much more work was needed to understand the implementation of care clusters and the development of payment systems based on them; a key need being robust analysis of cluster data (see, for example, NHS Confederation 2011; Clark 2011; Jacobs 2014).

With the sustained focus on the implementation of the cluster payment approach over a number of years, there has only recently been a more substantial quantity of care cluster data available to undertake analysis of the categorisation of patients. We contribute to the need for more robust analysis of clusters. In this paper we examine nationally available cluster data and consider how well the approach to clustering is operating, and reflect on the feasibility of its further development as a basis for episodic payment in mental health care.

The potential move towards an episodic payment system (Khan et al. 2014), would align mental health services more closely to the payment system used for acute physical health care in England, formerly called payment by results (PbR) but now known as the national tariff payment system (NTPS). Potential benefits of an episodic-based payment approach are that it increases transparency and accountability (The Mental Health Taskforce 2016), can incentivise providers to control unit costs, and deliver more efficient care (Jacobs 2014).

There is international interest in developing new forms of payment systems for secondary mental health care but experiences have been mixed. Australia and New Zealand developed casemix classification systems specific to mental health that incorporated information on patient severity and functioning using the Health of the Nation Outcome Scales (HoNOS). In both countries providers were shown to exhibit cost variations that rendered the classification systems unsuitable for payment, although this was an explicit objective in Australia only (Buckingham et al. 1998; Gaines et al. 2003). Some countries, such as the Netherlands, have included psychiatric care in the prospective activity-based payment system used for physical acute care (Kobel et al. 2011). This system takes account of the type of care and treatment provided as well as diagnosis (Forti et al. 2014). Cost control is incentivised by nationally agreed unit prices and the system also incentivises quality improvements that lead to lower resource consumption (Swan-Tan et al. 2011). Other countries have implemented prospective payment but chosen an alternative payment unit to the diagnosis casemix groups used for acute physical health care, such as in the United States which reimburses psychiatric inpatient care under Medicare using a per diem system, reflecting the fact that length of stay reflects cost (Mason and Goddard 2009).

In contrast to standard casemix systems which fix a patients’ categorisation and the payment that will be received for their treatment, under the episodic payment approach in England, patients are categorised into one of 21 care clusters according to their need and these are reviewed at set intervals. We describe the cluster model in more detail under “Methods” section.

Providers of services would be paid for the care a patient receives whilst assigned to a cluster for a defined review period or episode of care. Episodic payment therefore links a provider’s payment closely to the volume and type of mental health care *activity* that it supplies in such a way that the provider would know in advance how much each patient-cluster-period will yield it in terms of income (NHS England and NHS Improvement 2016) but nevertheless builds in flexibility as patients are reviewed. Fixed prices, agreed locally between commissioners and providers, are set for each cluster-episode. Alternatively, national average costs can be used to derive a national prospective fixed price.

Ultimately, an agreed payment rate needs to consider how prices relate to costs and the potential (perverse) incentives for care delivery (Jacobs 2014). A prospective fixed-price mechanism has found favour in many health care systems especially in regard to paying for acute hospital care (O’Reilly et al. 2012) but remains limited in mental health care as it is more difficult to define and cost an episode of care due to the interplay of such factors as the diversity of diagnosis, and the chronic and fluctuating nature of much mental illness (Wolff et al. 2016; Jacobs et al. 2015).

A key consideration for the viability of the episodic payment approach is in limiting the variation in costs associated with the defined payment categories. Cost variation needs to be considered both among patients *within* a given category or cluster and *between* providers for a given cluster. In considering the appropriate unit of activity for mental health services, the primary consideration is cost variation within the unit of activity since if this variation was minimal then all providers would produce at the same cost. However, for a given specification of categories, both forms of variation are important and if either is too great, problems may ensue. First, if a cluster captures a very diverse group of patients, there are a number of risks associated with setting a single price. Patients within the group who are exceptionally costly to treat are potentially loss-making to the provider. There is an incentive to treat only cheaper patients (“cream skimming”), refer more costly ones to other providers (“dumping”), or to reduce the treatments given to try and contain their cost (“skimping”) (Jacobs 2014). Unnecessarily moving people to another cluster in order to obtain higher income (cluster “creep”), is another option. Hence, excessive variation of cost within a treatment group is concerning (Moscelli et al. 2019).

The impact of variability of cost between providers is not straightforward. On the one hand a fixed price acts as an incentive for high-cost providers to control their costs so that *ex ante* variability in costs may not be a great concern in the medium to longer term. Alternatively, variation in costs across providers might indicate variation in their case mix and setting a uniform price based on average costs across providers might drive high cost, high quality providers who treat difficult patients into financial distress.

Therefore, for an episodic payment system to work, there should not be too much variation in costs *between* providers, or *within* clusters. There needs to be reasonable resource homogeneity within groups (both in terms of activity and costs) i.e. clusters should comprise patients who are homogenous in terms of resource use and ultimately cost. Some observed variation could be the result of poor data quality, and some may be due to legitimate variation in patient care. Either way, excessive variation in activity within each cluster will drive very high cost variation and hinder translation into a prospective cluster price, which will make the operation of an episodic payment approach difficult.

### Purpose of the Study

Using data on cluster assignments, costs and activity for all patients in contact with secondary mental health care services in England in the financial year 2014/15 we examine variation in costs and activity *within* clusters and *between* providers. We make three specific contributions. First, we provide an analysis of the type and variation of activity undertaken within care clusters, for example, the contacts with health care professionals which patients have. Second, we provide an assessment of how well clusters operate as a unit of activity in terms of categorising patients into resource homogenous groups. Third, by using the national administrative data which is intended to underpin the payment approach, we provide a comprehensive assessment of the proposed episodic payment system for England. Our results enable key policy lessons to be drawn for the future development of the payment approach.

## Methods

### Data

We use two main sources of data, the Mental Health Minimum Data Set (MHMDS) (Health & Social Care Information Centre) and Reference Costs (Department of Health 2015), for the financial year 2014/15.^1^ The MHMDS is a patient-level dataset with national coverage of all secondary mental health care for England and contains demographic, diagnostic and treatment information. The MHMDS contains data on the items of the Mental Health Clustering Tool (MHCT) which is used by a clinician or clinical team to assign a patient to one of 21 care clusters following guidance for scores on MHCT items (Monitor and NHS England 2013b). The MHCT consists of 18 items; the 13 items of the Health of the Nation Outcomes Scales (HoNOS) (Wing et al. 1994) and the five items of the Summary Assessment of Risk and Need (SARN) (Self et al. 2008, Self and Painter 2009). These are used to assess a patient’s need on both a current and historical basis. Each item is rated by staff on a scale of 0, no problems, to 4, severe to very severe problems. Clinicians or clinical teams then translate these scores according to guidance (Self et al. 2008, Self and Painter 2009) into assignments to the 21 clusters which reflect assessments of specific symptoms as well as needs and chronicity. Conceptually, these clusters are grouped into three super-clusters: non-psychotic (clusters 1–8), psychotic (clusters 10–17), and organic (clusters 18–21) (see Table 1). Cluster 9 is designated blank and not used, and 0 is a variance cluster, used to code someone on a short-term basis who cannot at that time be classified into one of the other 20 clusters but who does need some mental health treatment/support (Monitor and NHS England 2013a).

**Table 1:** Super-classes, clusters and review periods

Superclass	Cluster number	Cluster label	Cluster review period (maximum)
Variance cluster	0	Variance	6 months
Non-psychotic	1	Common mental health problems (low severity)	12 weeks
	2	Common mental health problems	15 weeks
	3	Non-psychotic (moderate severity)	6 months
	4	Non-psychotic (severe)	6 months
	5	Non-psychotic (very severe)	6 months
	6	Non-psychotic disorders of overvalued Ideas	6 months
	7	Enduring non-psychotic disorders (high disability)	Annual
	8	Non-psychotic chaotic and challenging disorders	Annual
N/A	9	Blank cluster	N/A
Psychosis	10	First episode in psychosis	Annual
	11	Ongoing recurrent psychosis (low symptoms)	Annual
	12	Ongoing or recurrent psychosis (high disability)	Annual
	13	Ongoing or recurrent psychosis (high symptom and disability)	Annual
	14	Psychotic crisis	4 weeks
	15	Severe psychotic depression	4 weeks
	16	Dual diagnosis (substance abuse and mental illness)	6 months
	17	Psychosis and affective disorder difficult to engage	6 months
Organic	18	Cognitive impairment (low need)	Annual
	19	Cognitive impairment or dementia (moderate need)	6 months
	20	Cognitive impairment or dementia (high need)	6 months
	21	Cognitive impairment or dementia (high physical need or engagement)	6 months

This table combines information from Table 1 (clusters and their review periods) and Figure 1 (super-classes and Clusters) from the “Mental health currencies and payment: 2014/15 guidance” (Monitor and NHS England 2013a)

The intention for these clusters was to group patients according to similar symptoms and needs and that these would be associated with similar sets of interventions or care packages (Royal College of Psychiatrists 2014). The clusters were developed according to a classification system of service users based on the similarity of their current needs and the similarity of their care plans (Self and Painter 2009).

*Cluster*-*episodes* are periods of time a patient spends assigned to the same cluster and these periods can comprise *admitted* (inpatient care) and *non*-*admitted* days (e.g. outpatient attendances, contacts with health care professionals in the community). The clusters are mutually exclusive meaning that a patient should only be assigned to one at any given time.

The system requires patients to be reviewed and re-assigned to clusters or discharged according to cluster review periods (see Table 1). If a person’s condition deteriorates and needs increase, they can be moved to a higher need cluster before the end of the review period of their current cluster. They should not be allocated to lower need clusters before designated review periods if their condition improves before then. National guidance specifies the fixed review periods for each cluster, though the actual duration of cluster-episodes does not necessarily reflect the review periods listed in Table 1, since after a review for a cluster-episode it can be decided that the patient should continue in the same cluster.

The MHMDS also records the activity which takes place while a patient is assigned to a Cluster. We focus on two types of activity: days spent admitted to hospital and days when the patient had contact with a health care professional. We remove observations where the number of admitted days is greater than the length of the cluster-episode. We count days with contact with a health care professional rather than all health care contacts because we observe cases with several contacts with health care professionals in the same day, which up to a point is feasible, for example a patient could have an appointment with two different types of staff, but is unlikely to have more than ten contacts on a day, which is observed for a small number of patients.

Reference Costs are a mandatory national data return for NHS providers in which they report their costs by cluster and by admitted and non-admitted days. These two cost measures (relating to admitted and non-admitted days) are available both at provider level and national level, with the latter reflecting the average of the costs reported by all providers.

### Statistical Analysis

We undertake two analyses. First, we analyse the variation *between* mental health providers in terms of their costs and activity. Second, we analyse the variation *within* clusters in terms of costs and activity.

Our unit of analysis in both cases is the cluster-episode, which records the time a patient spends assigned to one cluster.^2^ For each cluster-episode we observe its cluster number (see Table 1), its length ($epidays_{c}$) and the activity that takes place during it. We focus on two types of activity: length of stay or days spent admitted to hospital ($warddays_{c}$) and days when the patient had contact with a health care professional ($hpcondays_{c}$). The cluster number is used to match the corresponding (provider level and national average) costs, for admitted and non-admitted days, to each cluster-episode.

The *cost* for each cluster episode can be calculated as the sum of two costs, one for admitted days and the other for non-admitted days:

1 $$C_{c} = warddays_{c} *rc_{adm,c} + nonadm_{c} *rc_{nonadm,c}$$

where $warddays_{c}$ is the number of admitted days and $nonadm_{c}$ is the number of non-admitted days while assigned to cluster $c$, $rc_{adm,c}$ is the cost of an admitted day and $rc_{nonadm,c}$ is the cost of a non-admitted day in cluster $c$ obtained from Reference Costs. Since we have two types of cost, one at provider level and a national average, we can calculate two versions of the cost described in Eq. (1) for each provider, one based on the provider’s own costs and one based on a national average cost (i.e. a notional fixed price which could be used under an episodic payment approach).

The provider-level cost and the national average cost in turn can be used to calculate a cost index for each provider.

2 $$\frac{{\mathop \sum \nolimits_{\forall c} C_{c}^{p} }}{{\mathop \sum \nolimits_{\forall c} C_{c}^{NA} }} = \frac{{\mathop \sum \nolimits_{\forall c} \left( {warddays_{c} *rc_{adm,c}^{p} + nonadm_{c} *rc_{nonadm,c}^{p} } \right)}}{{\mathop \sum \nolimits_{\forall c} \left( {warddays_{c} *rc_{adm,c}^{NA} + nonadm_{c} *rc_{nonadm,c}^{NA} } \right)}}$$

where $warddays_{c}$ is the number of days spent as an inpatient and $nonadm_{c}$ is the number of non-admitted days while assigned to cluster $c$. Costs differ in their superscript, $p$ indicates provider level and $NA$ national average, $rc_{adm,c}$ is the cost of an admitted day and $rc_{nonadm,c}$ is the cost of a non-admitted day in cluster $c$. The index is then the provider-level average cluster costs where values of the index above 1 represent high cost providers while those below 1 are relatively low cost providers.

When analysing the variation in activity within clusters, we investigate whether longer cluster-episodes of care translate into proportionally more activity. This is relevant because activity drives costs and if activity is not proportional to the length of a cluster-episode the payment for a period of care would have to vary over the treatment period (e.g. pay more at the start of the treatment than at the end). Large within-cluster variation in activity could indicate that clusters are not resource homogenous. We use multilevel regressions (Rabe-Hesketh and Skrondal 2008) to reflect the hierarchical nature of the data. For all clusters, we consider a three-level model, where cluster-episodes are nested within patients and patients are nested within providers and report the results as elasticities, i.e. as the proportional change in activity for a proportional change in the length of the cluster-episode.

We regress the number of days with activity (i.e. ward days and contacts with health care professionals) on the length of a cluster-episode to obtain the correlation between the length of the cluster-episode and the activity performed in it. In both cases, the explanatory variable is the length of the cluster-episode. If all providers delivered the same services per period of time in a cluster (for example, 1 year), but reported it at different intervals (for example, quarterly or bi-annually), we would expect longer cluster-episodes to translate into proportionally more services delivered e.g. more contacts with health care professionals in comparison to shorter cluster-episodes. These regression coefficients are reported as elasticities and if activity within cluster-episodes is proportional to their length, we anticipate elasticities to be around one, i.e. longer (shorter) cluster-episodes of care translate into proportionally more (less) activity.

Equation (3) shows the regression for admitted days ($warddays_{cij}$). The explanatory variable is the length of the cluster-episode ($epidays_{cij}$), clusters ($c$) are nested within patients ($i$) and patients are nested within providers ($j$). We do not consider explanatory variables at the patient or provider levels, but these levels are reflected in the random intercepts $\zeta_{ij}^{\left( 2 \right)}$ for patient-provider combinations and $\zeta_{j}^{\left( 3 \right)}$ for providers, which are assumed to have a mean of zero. The regression for days with contact with a health care professional ($hpcondays$) is identical to Eq. (3) except for a switch in the dependent variable.

3 $$warddays_{cij} = \beta_{0} + \beta_{1} *epidays_{cij} + \epsilon_{cij} + \zeta_{ij}^{\left( 2 \right)} + \zeta_{j}^{\left( 3 \right)}$$

As a sensitivity analysis, we restrict the sample to finished (with recorded end date) cluster-episodes where patients can be either discharged from care or moved to another cluster-episode. We also estimate the model using only two levels (cluster-episodes nested within providers). All statistical analyses were carried out in Stata 13 (StataCorp. 2013).

## Results

### Descriptive Statistics

Table 2 shows the descriptive statistics for our sample. Note that the number of observations for the provider reported costs (superscript $p$) is smaller than for the national average costs (superscript $NA$). This is because the provider level costs were not available for all providers but we can still match the national averages to their activity.

**Table 2:** Descriptive statistics

Variable	Description (unit of measurement)	Obs.	Mean	Median	Std. Dev.	Min	Max
$\varvec{epidays}_{\varvec{c}}$	Days in cluster (days)	988,648	109.3	86.0	91.3	1.0	365.0
$\varvec{warddays}_{\varvec{c}}$	Days admitted to hospital (days)	988,648	2.5	0.0	13.5	0.0	364.0
$\varvec{rc}_{{\varvec{adm},\varvec{c}}}^{{\varvec{NA}}}$	National average cost admitted days (£)	988,648	361.9	360.0	18.4	324.2	396.4
$\varvec{rc}_{{\varvec{nonadm},\varvec{c}}}^{{\varvec{NA}}}$	National average cost non-admitted days (£)	988,648	9.2	8.1	5.9	3.4	37.3
$\varvec{rc}_{{\varvec{adm},\varvec{c}}}^{\varvec{p}}$	Provider level cost admitted days (£)	967,848	365.6	358.7	90.3	6.3	1,456.5
$\varvec{rc}_{{\varvec{nonadm},\varvec{c}}}^{\varvec{p}}$	Provider level cost non-admitted days (£)	985,139	10.1	8.6	10.6	0.2	427.5
$\varvec{hpcondays}_{\varvec{c}}$	Days with contact with a health care professional (days)	988,648	6.6	3.0	10.3	0.0	356.0

Not all providers report Reference Costs for all the Clusters, therefore the cost variables at provider level have fewer observations than the national ones. All costs are for the financial year 2014/15

Patients spent on average 3½ months in a cluster, with 2 days as an inpatient on a ward and had contact with a health care professional for 7 days. On average, cost for inpatient care was around £365 per day whilst care in the community or as an outpatient was £10 per day. It is evident from Table 2 that there is a huge amount of variation between providers in terms of costs, activity and length of stay.

Descriptive statistics for each cluster are shown in Table 3. It shows the distribution of the cluster-episodes across the different care clusters. The largest are clusters 4, 18 and 19 with around 12% of cluster-episodes, while cluster 3 has around 9% of cluster-episodes allocated to it.

**Table 3:** Descriptive statistics by cluster

Cluster	Cluster-episodes		$\varvec{epidays}_{\varvec{c}}$ (days)		$\varvec{warddays}_{\varvec{c}}$ (days)		$\varvec{rc}_{{\varvec{adm},\varvec{c}}}^{{\varvec{NA}}}$ (£)		$\varvec{rc}_{{\varvec{nonadm},\varvec{c}}}^{{\varvec{NA}}}$ (£)		$\varvec{rc}_{{\varvec{adm},\varvec{c}}}^{\varvec{p}}$ (£)		$\varvec{rc}_{{\varvec{nonadm},\varvec{c}}}^{\varvec{p}}$ (£)		$\varvec{hpcondays}_{\varvec{c}}$ (days)	
	Number	%	Mean	Std. Dev.	Mean	Std. Dev.	Mean	Std. Dev.	Mean	Std. Dev.	Mean	Std. Dev.	Mean	Std. Dev.	Mean	Std. Dev.
0	11,563	1.17	74.9	85.3	0.8	7.3	324.2	0.0	4.3	0.0	309.4	116.0	8.1	7.5	3.2	5.4
1	21,291	2.15	50.6	65.6	0.6	4.2	346.9	0.0	5.7	0.0	353.4	147.8	8.3	4.6	2.6	4.0
2	26,647	2.70	69.7	76.4	0.6	5.3	329.0	0.0	6.3	0.0	334.1	136.6	7.7	4.7	3.1	5.1
3	86,234	8.72	90.5	83.6	0.9	6.8	345.4	0.0	7.4	0.0	341.1	77.5	7.9	3.7	4.4	7.0
4	114,109	11.54	103.8	84.5	1.2	8.6	345.8	0.0	8.7	0.0	350.0	65.0	9.0	3.1	6.3	8.5
5	48,274	4.88	95.0	85.5	3.1	14.2	342.5	0.0	12.5	0.0	348.7	55.5	13.6	4.4	8.2	10.6
6	22,451	2.27	113.1	87.1	1.7	10.7	342.7	0.0	10.4	0.0	349.6	62.2	11.1	4.1	8.0	10.2
7	54,671	5.53	124.6	97.6	2.0	12.2	347.8	0.0	9.8	0.0	353.5	70.5	10.1	3.0	8.3	11.4
8	48,131	4.87	104.0	94.2	2.9	13.4	369.1	0.0	13.7	0.0	363.3	75.9	14.1	4.6	9.3	13.1
10	27,553	2.79	113.9	95.4	4.9	17.9	361.6	0.0	17.2	0.0	355.4	59.9	17.8	5.3	11.5	13.7
11	65,760	6.65	131.8	99.7	1.9	11.8	348.9	0.0	8.1	0.0	356.9	58.0	8.6	2.5	8.0	11.1
12	59,551	6.02	133.6	100.6	4.0	17.9	369.3	0.0	11.7	0.0	365.9	73.7	12.3	3.7	10.7	14.3
13	36,559	3.70	124.1	99.5	8.8	27.5	357.6	0.0	15.3	0.0	365.6	62.8	16.1	6.2	12.5	16.3
14	21,724	2.20	44.2	56.1	12.9	21.2	396.4	0.0	37.3	0.0	400.9	91.8	49.8	43.8	8.3	10.6
15	5960	0.60	59.0	67.6	9.9	21.2	369.6	0.0	21.5	0.0	367.5	81.4	29.0	18.9	8.3	10.9
16	11,816	1.20	106.2	85.4	6.2	20.8	366.9	0.0	14.5	0.0	368.9	64.0	15.5	6.1	10.8	14.6
17	15,895	1.61	111.8	80.8	8.2	25.4	360.0	0.0	21.5	0.0	362.3	56.3	23.2	8.5	15.1	19.5
18	115,125	11.64	125.1	95.5	0.2	4.3	372.7	0.0	3.4	0.0	387.7	135.5	3.6	1.8	3.2	4.7
19	125,115	12.66	118.8	85.3	0.8	8.5	388.4	0.0	4.8	0.0	393.3	85.2	5.3	3.1	4.1	6.8
20	50,384	5.10	111.9	83.9	3.8	18.7	389.8	0.0	5.7	0.0	388.3	101.6	6.1	3.2	4.8	8.0
21	19,835	2.01	106.9	83.7	3.9	19.3	383.2	0.0	5.7	0.0	387.7	91.4	6.0	3.0	4.2	7.3

For variable definitions see Table 2

#### Cost Index

Table 4 shows the cost index, as calculated in Eq. (2), for each provider. We observe that the highest-cost provider is 58% above average and the lowest-cost provider is 27% below average, resulting in a ratio between the maximum cost and the minimum cost of 2.16.

**Table 4:** Cost Index at provider-level

Provider	Cost in £000		Cost Index	Provider	Cost in £000		Cost Index
	Provider-level	National average			Provider-level	National average	
1	26,975	17,033	1.58	30	20,059	19,304	1.04
2	40,690	29,037	1.40	31	26,361	25,421	1.04
3	78,502	59,910	1.31	32	21,288	20,570	1.03
4	45,275	35,958	1.26	33	26,314	25,562	1.03
5	60,469	48,157	1.26	34	66,783	65,472	1.02
6	3612	2955	1.22	35	36,994	36,354	1.02
7	16,342	13,461	1.21	36	33,322	32,783	1.02
8	43,626	36,033	1.21	37	27,527	27,414	1.00
9	25,345	21,162	1.20	38	20,163	20,222	1.00
10	42,652	36,891	1.16	39	34,682	34,827	1.00
11	6550	5721	1.15	40	22,738	23,244	0.98
12	8724	7676	1.14	41	18,405	18,817	0.98
13	24,980	22,170	1.13	42	18,903	19,372	0.98
14	45,197	40,395	1.12	43	12,880	13,230	0.97
15	29,514	26,682	1.11	44	31,117	32,618	0.95
16	12,589	11,431	1.10	45	73,666	77,863	0.95
17	66,282	60,226	1.10	46	62,504	67,029	0.93
18	16,333	14,881	1.10	47	25,382	27,736	0.92
19	42,916	39,223	1.09	48	25,453	28,024	0.91
20	29,982	27,433	1.09	49	33,198	36,892	0.90
21	55,295	50,950	1.09	50	31,469	36,250	0.87
22	16,433	15,290	1.07	51	9199	10,609	0.87
23	33,401	31,448	1.06	52	21,111	24,652	0.86
24	36,384	34,405	1.06	53	48,419	57,227	0.85
25	37,095	35,152	1.06	54	44,541	54,282	0.82
26	49,820	47,362	1.05	55	17,880	21,902	0.82
27	35,761	34,060	1.05	56	24,950	34,112	0.73
28	28,875	27,559	1.05	57	36,536	50,325	0.73
29	19,259	18,483	1.04				
Mean	32,469	31,461		Min	3612	2955	
St. Dev.	16,840	16,212		Max	78,502	77,863	

Cost index provides provider-level average cluster costs. Values of the index above 1 are relatively high cost providers while those below 1 are relatively low cost providers

Table 5 shows the maximum and minimum cost index for each cluster, and the ratio between them. We observe that the clusters where the difference between most and least expensive provider is greatest, are clusters 0, 1, 2 and 3 where the minimum index is < 25%, giving rise to variation within clusters of more than 20-fold between most- and least-cost providers.

**Table 5:** Average cluster-episode cost and cost index summary by cluster

	Number of providers	Average cluster-episode cost (National average) [£)	Minimum as % of National average	Maximum as % of National average	Maximum/minimum
Cluster 0	48	571	24	490	20.55
Cluster 1	50	487	11	335	31.88
Cluster 2	51	635	2	377	179.33
Cluster 3	53	958	8	191	23.47
Cluster 4	57	1316	56	229	4.09
Cluster 5	57	2221	32	162	5.00
Cluster 6	55	1729	20	173	8.78
Cluster 7	57	1882	46	160	3.50
Cluster 8	56	2459	37	175	4.73
Cluster 10	57	3655	45	155	3.40
Cluster 11	55	1734	56	176	3.15
Cluster 12	57	2980	63	183	2.93
Cluster 13	56	4896	68	152	2.23
Cluster 14	57	6290	68	420	6.22
Cluster 15	56	4721	44	225	5.12
Cluster 16	55	3739	52	169	3.22
Cluster 17	56	5171	55	155	2.81
Cluster 18	55	512	42	284	6.76
Cluster 19	57	885	41	236	5.78
Cluster 20	57	2080	57	218	3.83
Cluster 21	56	2081	35	167	4.83

Maximum and minimum cost index for each cluster across all providers

#### Activity Regressions

Table 6 shows the multi-level model regression results for ward days and Table 7 for days with contact with health care professionals. The results are based on the regression of Eq. (3), using the two different dependent variables (ward days and contacts with health care professionals), employing a post-estimation command in Stata (*margins*) to obtain the results as elasticities. For both dependent variables all elasticities are significant and smaller than one, which indicates that longer cluster-episodes do not translate into proportionally more activity.

**Table 6:** Three-level regression model for ward days

	Elasticity	Std. Err.	*p* value	N
Cluster 0	0.2418	0.0501	< 0.01	11,563
Cluster 1	0.1568	0.0315	< 0.01	21,291
Cluster 2	0.2267	0.0374	< 0.01	26,647
Cluster 3	0.1449	0.0250	< 0.01	86,234
Cluster 4	0.1410	0.0214	< 0.01	114,109
Cluster 5	0.2174	0.0215	< 0.01	48,274
Cluster 6	0.1422	0.0459	< 0.01	22,451
Cluster 7	0.1681	0.0292	< 0.01	54,671
Cluster 8	0.1858	0.0200	< 0.01	48,131
Cluster 10	0.2199	0.0228	< 0.01	27,553
Cluster 11	0.1167	0.0260	< 0.01	65,760
Cluster 12	0.1369	0.0197	< 0.01	59,551
Cluster 13	0.3119	0.0224	< 0.01	36,559
Cluster 14	0.3040	0.0136	< 0.01	21,724
Cluster 15	0.2417	0.0214	< 0.01	5,960
Cluster 16	0.3293	0.0342	< 0.01	11,816
Cluster 17	0.3827	0.0365	< 0.01	15,895
Cluster 18	0.2219	0.0549	< 0.01	115,125
Cluster 19	0.2393	0.0404	< 0.01	125,115
Cluster 20	0.3870	0.0407	< 0.01	50,384
Cluster 21	0.3957	0.0512	< 0.01	19,835

Ward days is the dependent variable. Length of cluster-episode is the explanatory variable. Levels are cluster-episodes—patient—provider. Elasticity = (proportional change in ward days)/(proportional change in length of cluster-episode)

**Table 7:** Three-level regression model for days with contact with a health care professional

	Elasticity	Std. Err.	p-value	N
Cluster 0	0.3589	0.0250	< 0.01	11,563
Cluster 1	0.3105	0.0133	< 0.01	21,291
Cluster 2	0.3700	0.0141	< 0.01	26,647
Cluster 3	0.4648	0.0176	< 0.01	86,234
Cluster 4	0.4957	0.0208	< 0.01	114,109
Cluster 5	0.4676	0.0186	< 0.01	48,274
Cluster 6	0.6198	0.0278	< 0.01	22,451
Cluster 7	0.6180	0.0282	< 0.01	54,671
Cluster 8	0.6181	0.0321	< 0.01	48,131
Cluster 10	0.6758	0.0378	< 0.01	27,553
Cluster 11	0.6298	0.0264	< 0.01	65,760
Cluster 12	0.6288	0.0293	< 0.01	59,551
Cluster 13	0.6208	0.0311	< 0.01	36,559
Cluster 14	0.3827	0.0235	< 0.01	21,724
Cluster 15	0.3663	0.0233	< 0.01	5,960
Cluster 16	0.6381	0.0430	< 0.01	11,816
Cluster 17	0.8204	0.0793	< 0.01	15,895
Cluster 18	0.5004	0.0219	< 0.01	115,125
Cluster 19	0.4613	0.0233	< 0.01	125,115
Cluster 20	0.4571	0.0269	< 0.01	50,384
Cluster 21	0.4232	0.0322	< 0.01	19,835

Days with contact with a health care professional is the dependent variable. Length of cluster-episode is the explanatory variable. Levels are cluster-episodes—patient—provider. Elasticity = (proportional change in days with contact with a health care professional)/(proportional change in length of cluster-episode)

The results are robust to the sensitivity analyses that restricted the sample to include only finished cluster-episodes and used a two-level model (cluster-episodes nested within providers).

## Discussion

### Contribution to the Current Evidence Base

This paper has explored the proposed episodic payment approach for mental health services in England whereby clinicians allocate patients into one of 21 clusters on the basis of similar levels of need using the MHCT. For this episodic payment system to work effectively, there should not be too much variation in costs or resource use either *within* clusters, or *between* providers. We tested whether the existing unit of activity, namely clusters, which underpin the collection of mental health activity and cost data amongst English mental health providers, would support the new payment system. Specifically, we examined the variation both within clusters and between mental health providers in terms of their costs and activity/resource use.

We contribute to the evidence base by examining the implementation of care clusters as a unit of activity in mental health, with a key need being more robust analysis of cluster data. Our results suggest a large amount of variation between providers in terms of costs, activity rates and length of stay within clusters. There is substantial variability between providers in the length of cluster episodes, and there is huge variability within clusters in terms of the proportion of inpatient days and the proportion of contact with health care professionals. We find longer cluster episodes do not translate into proportionally more activity in terms of either inpatient days or contacts with health care professionals. With high levels of variation within clusters, accurate baseline activity rates cannot be determined for planning and purchasing care. Variation in activity rates means that providers see different numbers of patients, have different treatment approaches, levels of productivity, and put different care pathways and packages of care in place for patients within each cluster. This could lead to differences in care quality and outcomes across providers, generating potential geographic inequalities for patients. While the average costs per cluster (Table 5) broadly correspond to severity as indicated by the cluster labels (Table 1), there is also enormous variation within clusters in terms of costs. Variations in cost mean that patients with similar levels of need may be using different levels of resource, leading to a potential waste of scarce resources or an under-treatment of some people in some localities. This also suggests that the introduction of an episodic payment approach would result in large variation across providers in terms of their financial positions.

Our findings of significant heterogeneity in costs, and significant heterogeneity in terms of resource use, do not bode well for an episodic payment approach which requires resource homogeneity within clusters. The reduction of variation in care, activity levels and costs is pivotal to the establishment of a well-designed classification and payment system.

Of course, some of the variation we have found could be a result of data quality issues, including poor costing systems, poor coding, and differing allocation of patients to clusters between individual clinicians and providers. Even if this is the case, it still does not augur well for an episodic payment system since until the data quality issues are resolved, the analysis underpinning it will result in inappropriate payments to providers.

### Policy Implications

Whilst the findings from our study appear discouraging for the prospect of an episodic payment approach in mental health, we would argue that instead of abandoning the episodic payment approach and clustering altogether, a much clearer steer is needed from policymakers to support providers and commissioners to move towards refining and developing episodic payment as a viable payment option. A step in this direction would be increasing investment in information technology and underpinning data systems (such as outcomes and patient level costing systems). The improvement of data quality needs to be a priority in mental health services, since without robust evidence regarding the incurred costs, building any kind of payment system is an impossible task.

Another step could be re-designing or refining the clusters to improve homogeneity, and indeed there may be a case for increasing the number of clusters to make them more resource homogenous. In the acute hospital sector it took more than a decade since its implementation to refine and develop the Payment by Results approach (Appleby et al. 2012), and a similar development and refinement period could be anticipated for mental health services to ensure clusters are improved over time and become fit for purpose. Indeed the number of HRGs has increased exponentially over time as part of this refinement process. There has been significant investment in staff development, information technology and collection of cost and other data over time which the acute hospital sector has benefited from and is still learning how to gain even more from (Marini and Street 2007).

The system also needs to implement change at a pace that does not risk destabilising local health economies and that fits with other priorities and developments in health and social care policy nationally and locally. Having said that, the slow movement within the sector around transitioning to alternative payment approaches and the current policy focus on devolving more decisions to local levels, means that this risk is quite low. These developments also need to bear in mind the evidence that developing and implementing new payment models is a long, complex process (ICF Consulting Services 2015).

### Limitations and Future Research

Our research shows that it will be difficult to create a classification and payment system with the currently available data (MHMDS). A key source of the variation we have identified is driven by poor quality data in the MHMDS e.g. duplicate data, missing data such as end dates, overlapping cluster-episodes for the same patient, all which require significant effort and expertise to clean and use. We have specifically limited our analysis to measures of resource use and activity which could be reliably defined to ensure the robustness of our results. However, the data quality would improve significantly if commissioners required all providers to use the MHMDS dataset for contracting and payment, rather than producing their own spreadsheets for such purposes, and this would facilitate greater national benchmarking and expanded research opportunities.

The cost data in particular are of a relatively poor quality and cannot be used at present to identify a reliable pricing system. For the development of any payment system, high quality activity and cost data would be a key requisite. Data quality is a significant challenge with any payment system, but it is at least underway for the episodic payment approach using clustering, collected routinely and there is evidence of some improvements in data quality over time in its collection (Jacobs et al. 2016). Improvements to reference cost data are also essential and the introduction of patient level information costing systems (PLICS) at provider level can support the process of generating this. Future research may explore the advantages in robustness that patient level costing systems could provide compared to cluster level costing and whether these reduce variation within clusters. It would also be valuable for future research to compare and contrast the pros and cons of different units of activity used in payment systems internationally so that policymakers can learn from the design of different payment approaches.

## Conclusion

Our analysis of cost and activity for all secondary mental health care in England in the financial year 2014/2015 has revealed substantial variability between providers as well as within clusters. While such variability in and of itself does not make efficient pricing arrangements impossible, our results indicate that a cluster-based episodic payment system would have substantially different financial impacts across providers which could destabilise local health economies. Our analysis further suggests that the inconsistent quality of the currently available mental health care cost and utilization data in England will make it difficult to create a fair and accurate episodic pricing system for mental health care. Greater investment in developing more accurate and consistent information and costing systems for health care may be needed to support such episodic payment systems in the future.

## Footnotes

1 In September 2014 the MHMDS was replaced by the Mental Health and Learning Disabilities Dataset (MHLDDS), therefore the data for the financial year 2014/15 comes from these two datasets.

2 Cluster-episodes can go over two financial years which run from 1 April to 31 March (e.g. a cluster-episode can start in January and end in May). We consider cluster-episodes that start during the financial year 2014/15, and for cluster-episodes that did not record an end date, we impute as end date the end of the financial year, i.e. on 31st March 2015.
